# A Retrospective Study on the Clinicopathological Characteristics and Prognostic Analysis of Gynecologic Neuroendocrine Carcinoma

**DOI:** 10.1002/cam4.71488

**Published:** 2025-12-31

**Authors:** Yedan Ren, Sen Li, Simin Xiang, Junfen Xu

**Affiliations:** ^1^ Department of Gynecology and Oncology Women's Hospital, Zhejiang University School of Medicine Zhejiang Hangzhou China; ^2^ Zhejiang Key Laboratory of Precision Diagnosis and Therapy for Major Gynecological Diseases Women's Hospital, Zhejiang University School of Medicine Zhejiang Hangzhou China; ^3^ Zhejiang Provincial Clinical Research Center for Obstetrics and Gynecology Zhejiang Hangzhou China

**Keywords:** FIGO stage, gynecological neuroendocrine carcinoma, lymph node metastasis, MiNEN, survival analysis

## Abstract

**Background:**

Gynecologic neuroendocrine carcinomas (NECs) are rare, highly aggressive malignancies with early metastatic potential and limited evidence to guide optimal management across different primary sites.

**Aims:**

To characterize the clinicopathological features, treatment patterns, survival outcomes, and prognostic factors of gynecologic NECs (cervix, endometrium, and ovary) treated at a single tertiary center over a 10‐year period.

**Materials and Methods:**

This observational, single‐center retrospective cohort study included patients diagnosed with gynecologic NEC at Women's Hospital, Zhejiang University School of Medicine, between January 2013 and August 2023. Clinicopathological data, treatment modalities, recurrence, and follow‐up outcomes were collected. Progression‐free survival (PFS) and overall survival (OS) were estimated using Kaplan–Meier methods. Prognostic factors were assessed using log‐rank tests and multivariable Cox proportional hazards models.

**Results:**

A total of 52 patients were identified, of whom 78.8% had cervical NEC. Primary surgery was performed in 90.4% of patients; adjuvant chemotherapy and radiotherapy were administered in 65.4% and 51.9%, respectively. Among cervical NEC cases with HPV testing, 69.7% were HPV16/18‐positive. Immunohistochemical (IHC) showed high positivity for synaptophysin (95.3%) and chromogranin A (72.7%); Ki‐67 exceeded 50% in 89.1% of evaluated cases. Median PFS for cervical NEC was 29 months; stage I cervical NEC showed a 5‐year PFS of 51.6% and 5‐year OS of 68.4%. Poorer prognosis was associated with FIGO stage ≥ IB3, mixed neuroendocrine‐non‐neuroendocrine neoplasm (MiNEN) with squamous cell carcinoma, tumor size > 4 cm, and lymph node metastasis. On multivariable analysis of cervical NEC, MiNEN with squamous cell carcinoma remained an independent predictor of reduced PFS (HR = 6.97, 95% CI: 1.60–30.31; *p* = 0.010).

**Discussion:**

Despite multimodal treatment, gynecologic NECs showed poor outcomes. The identification of MiNEN with squamous cell carcinoma as an independent adverse factor for PFS suggests histologic composition may meaningfully affect prognosis and warrants validation in larger, multicenter cohorts.

**Conclusion:**

Cervical NEC was the predominant subtype, most patients underwent surgery with adjuvant therapy, and survival was strongly stage‐dependent. MiNEN with squamous cell carcinoma independently predicted worse PFS, highlighting a potential high‐risk subgroup and reinforcing the need for multicenter prospective studies and more effective, potentially targeted treatment approaches for gynecologic NECs.

## 1 Introduction

Neuroendocrine carcinomas (NECs) are rare, highly aggressive malignancies arising in diverse anatomical sites, including the lungs, pancreas, gastrointestinal tract, and gynecologic organs [1]. Among subtypes of gynecologic NEC, cervical NEC is the most common, accounting for approximately 1.4% of all malignant cervical tumors [2]. Ovarian NECs represent only 1%–2% of all ovarian carcinomas, with approximately 100 cases documented in the literature [3]. The incidence at other gynecologic sites, such as the vulva, is even lower [4].

Despite their rarity, gynecologic NECs are associated with an aggressive clinical course characterized by early metastasis and a poor prognosis [4, 5]. Current treatment strategies are largely adapted from protocols for other malignant neuroendocrine tumors or more prevalent gynecologic cancers. These strategies typically involve multimodal therapy, including surgery, radiation, chemotherapy, targeted therapy, and immunotherapy [6]. However, a consensus on the most effective treatment regimen is lacking because of the limited number of studies and the heterogeneous nature of patient populations.

For patients with early‐stage cervical NEC, the primary treatment involves a combination of radical surgery and adjuvant chemotherapy, typically with etoposide and cisplatin [7]. For locally advanced or recurrent cervical NEC, combined radiochemotherapy is recommended [6]. In cases of ovarian NEC, debulking surgery followed by adjuvant chemotherapy, primarily with carboplatin/paclitaxel or etoposide/cisplatin, is preferred [8]. In patients with early‐stage and advanced endometrial NEC, surgery is often supplemented with platinum‐based chemotherapy and/or radiotherapy [9, 10]. The 5‐year overall survival (OS) rate varies significantly by anatomical site and disease stage at diagnosis; for example, early‐stage cervical NEC has a 5‐year OS rate of 71%, which decreases to 36% for locally advanced disease and further decreases to 12% for advanced‐stage cases [11]. A retrospective study of 50 patients with ovarian neuroendocrine neoplasms reported a 5‐year OS rate of only 24% and a median survival of only 1.6 year [12]. In patients with endometrial NEC, small retrospective studies have shown a 5‐year OS rate of 29.4% for pure small‐cell neuroendocrine carcinoma (SCNEC) and 57.1% for pure large‐cell neuroendocrine carcinoma (LCNEC) [13].

The rarity of gynecologic NECs presents significant challenges in establishing evidence‐based guidelines. Furthermore, the molecular characteristics of these tumors remain insufficiently explored. Common genetic mutations and pathway alterations in NECs include frequent alterations in the PI3K/AKT/mTOR and RAS/MEK signaling pathways, with mutations in PI3KCA (41.2%), PTEN (35%), and KRAS/BRAF (11.3%) being particularly prevalent [14, 15, 16, 17]. Additionally, mutations in MYC, TP53, homologous recombination repair genes, and genes of the ERBB pathway are also commonly observed [17]. However, targeted therapies for these tumors are still in the early stages of exploration.

This study aimed to present the clinicopathological features, treatment options, OS rates, and prognostic factors of patients diagnosed with gynecological NECs at the Women's Hospital, Zhejiang University School of Medicine, between January 2013 and August 2023. The NECs in this study originated from the cervix, ovary, and endometrium. Through retrospective analysis, we found that gynecological NECs were primarily treated with surgery, complemented by active adjuvant therapy. Cervical NEC was the most prevalent form, whereas ovarian NEC was the rarest. In contrast to endometrioid uterine carcinoma, which is often diagnosed at earlier stages, endometrial NEC frequently presents at advanced stages, adversely impacting prognosis. This study underscores the need for a more comprehensive understanding of these rare malignancies and highlights the potential for targeted therapies to improve patient outcomes.

## 2 Methods

### 2.1 Data Collection

This was an observational, single‐center, retrospective cohort study including patients with gynecological NECs diagnosed between January 2013 and August 2023 at Women's Hospital, Zhejiang University School of Medicine. The inclusion criteria were as follows: (1) patients who were diagnosed with NEC of the gynecologic tract and (2) patients who were diagnosed between January 2013 and August 2023. The exclusion criteria were as follows: (1) patients who were diagnosed with primary NEC of an organ other than those of the gynecologic tract, (2) patients with a history of other types of cancer prior to the diagnosis of NEC, (3) patients presenting other types of cancers at diagnosis, (4) patients with low‐grade neuroendocrine tumors, (5) patients with carcinomas with neuroendocrine differentiation, and (6) patients with carcinoid tumors. Initially, we searched our institutional medical records system using the keyword “neuroendocrine”, which yielded 138 patient records. After patients with G1/G2 neuroendocrine tumors, breast NECs, carcinoid tumors, and tumors with only focal neuroendocrine differentiation were excluded, 80 patients met the preliminary criteria. We then excluded patients whose medical records were incomplete. In total, 52 patients with cervical, endometrial, or ovarian NEC were included in the analysis. The cervical carcinoma classifications under the 2009 or 2014 FIGO clinical staging systems were modified to meet the definitions of the 2018 FIGO clinical staging system.

The data collected included the age distribution, initial symptoms, laboratory results, pathological characteristics, treatment regimens, recurrence data, and survival outcomes of the eligible patients. The pre‐treatment inflammatory markers and nutritional index were calculated. The neutrophil‐lymphocyte ratio (NLR) was calculated as the ratio of the total neutrophil count to the total lymphocyte count. The Prognostic Nutritional Index (PNI) was calculated using the following formula: 10 × serum albumin (g/dL) + 0.005 × total lymphocyte count (per mm^3^). The NLR cutoff was determined using the Youden index of the receiver operating characteristic (ROC) curve to identify the optimal thresholds for predicting OS status. The PNI and lactate dehydrogenase (LDH) cutoffs were determined using the median value. The treatment protocols included surgery, chemotherapy, radiotherapy, or a combination of these modalities. Patients classified as having undergone surgery had at least one hysterectomy, which could be total, extrafascial, modified radical, or radical, with or without bilateral salpingo‐oophorectomy (BSO). The choice of surgical approach was based on the patient's disease stage, tumor location, and relevant NCCN guidelines for the year in which surgery was performed. Recurrence data and survival outcomes were determined based on follow‐up information obtained from medical records and/or patient self‐reports. OS and progression‐free survival (PFS) were further investigated. OS was defined as the time from the date of first diagnosis to the date of death, with patients who were last known to be alive at the date of last follow‐up censored. PFS was defined as the time from the date of first diagnosis to the date of the first documented disease progression or death, regardless of cause, and patients who were alive and progression‐free at the time of last follow‐up were censored.

This study received approval from the Medical Research Ethics Committee of the Women's Hospital, Zhejiang University School of Medicine.

### 2.2 Immunohistochemical Staining and Patient Diagnosis

NEC was primarily diagnosed through cell morphology analysis via hematoxylin and eosin (H&E) staining, along with immunohistochemical (IHC) analyses, which were conducted by two senior pathologists. The pathological criteria were defined according to the 2014 WHO classification of tumors of the female reproductive organs. According to the guidelines, SCNEC can be diagnosed primarily based on morphology without IHC confirmation, whereas LCNEC requires IHC evidence for diagnosis. IHC analysis was restricted to markers routinely assessed during clinical diagnosis in accordance with our institutional protocols. All suspected NEC cases were tested for at least two of the key neuroendocrine markers (synaptophysin [Syn], chromogranin A [CgA], CD56, and neuron‐specific enolase [NSE]), although marker positivity was not a mandatory criterion for diagnosis, which remained primarily morphology‐based. Additional IHC analysis is essential for differentiating NEC from other tumor types, including poorly differentiated carcinoma and metastatic small‐cell carcinoma. Evaluation of the following immunomarkers is also sometimes necessary: p16, p53, p40, p63, CAM5.2, CK5/6, CK20, E‐cadherin, thyroid transcription factor‐1 (TTF‐1), epidermal growth factor receptor (EGFR), melanoma, S‐100, human melanoma black 45 (HMB45), smooth muscle actin (SMA), and Ki‐67, among others. NECs were classified as “pure” if they consisted exclusively of neuroendocrine carcinoma. In contrast, carcinomas of the nonneuroendocrine type admixed with an NEC component were classified as mixed neuroendocrine‐non‐neuroendocrine neoplasms (MiNENs).

### 2.3 Treatment

The management of gynecologic NECs in this cohort was multimodal and included surgery, chemotherapy, and/or radiotherapy. In this study, surgery refers exclusively to radical procedures, as defined by the NCCN guidelines for the respective tumor sites. The chemotherapy regimens included etoposide/cisplatin, paclitaxel/cisplatin/bevacizumab, and paclitaxel/carboplatin.

### 2.4 Statistical Analysis

Survival and prognostic analyses were performed using SPSS version 23.0 and GraphPad Prism 9.5.0. Analysis of descriptive statistics was conducted with R version 4.4.2. Continuous variables are summarized as the means with standard deviations (SDs), whereas categorical variables are presented as frequencies and percentages. Group comparisons were performed by tumor site (cervical, ovarian, and endometrial NECs) and significance testing was carried out via ANOVA for continuous variables and Fisher's exact test for categorical variables. The variables included in the analysis were age, body mass index (BMI), FIGO stage, histological subtype, lymph node metastasis (LNM), treatment modality, and human papilloma virus (HPV) test results. The follow‐up period for all participants ranged from 1 to 134 m. PFS and OS were estimated via Kaplan–Meier analysis. Univariate analysis was conducted via the log‐rank test to identify potential prognostic factors. Variables with *p* < 0.05 in the univariate analysis were subsequently included in a multivariate Cox proportional hazards regression model. Statistical significance was set at *p* < 0.05 for all tests. The results of both univariate and multivariate analyses are presented as hazard ratios (HRs) with 95% confidence intervals (CIs) determined using the Cox proportional hazards regression model.

## 3 Results

### 3.1 Patient Demographics and Clinical Characteristics

**FIGURE 1:**
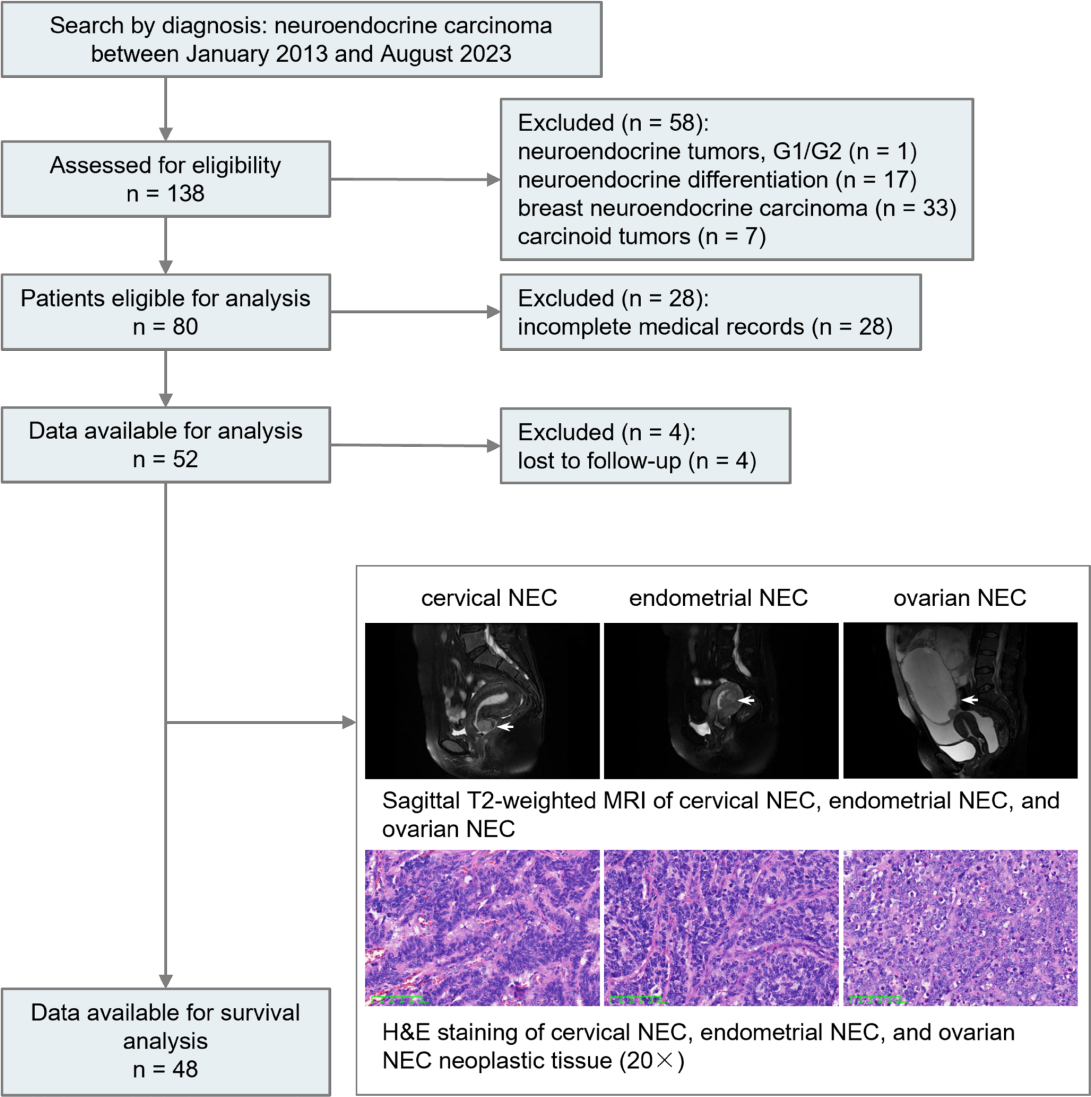
Flow diagram describing the process for patient inclusion and exclusion. G1/G2, Grade 1 or Grade 2; H&E staining, hematoxylin and eosin staining; MRI, magnetic resonance imaging; NEC, neuroendocrine carcinoma.

This observational, single‐center, retrospective cohort study included patients with gynecological NEC treated at the Women's Hospital, Zhejiang University School of Medicine, from January 2013 to August 2023. Initially, 138 patients were assessed, with 80 patients meeting the inclusion criteria after excluding those with neuroendocrine tumors G1/G2 (*n* = 1), breast NEC (*n* = 33), carcinoid tumors (*n* = 7), and neuroendocrine differentiation (*n* = 17). Eligibility was confirmed by pathological evidence, and patients with significant loss of medical records, those seeking pathology consultation from other medical institutions (*n* = 23) and those visiting other medical institutions for treatment (*n* = 5) were excluded. This left 52 patients for inclusion in the clinical characteristic analysis; 48 patients were included in the survival analysis, as 4 patients were lost to follow‐up (Figure 1). Clinical and pathological data were collected retrospectively for analysis.

The clinical characteristics of the patients at diagnosis are summarized in Table 1. Patient ages ranged from 26 to 74 year, with a mean age of 48 year. The clinical presentation of gynecological NECs was nonspecific, and postcoital vaginal bleeding (20/52) and irregular vaginal bleeding (20/52) were the most common symptoms. A smaller subset of cases (7/52) was detected incidentally during screening, and one rare case presented with abnormal vaginal discharge. Notably, no patients presented classic neuroendocrine symptoms. Among all patients, surgery was the most commonly used treatment and was performed in 47 patients (90.4%). Among the entire cohort, 59.1% of patients were diagnosed with stage I disease, but only two patients were clearly documented as not receiving additional adjuvant treatment. These findings indicate that although the majority of patients were diagnosed at an early stage, most patients underwent adjuvant therapies, including radiotherapy, chemotherapy, or a combination of both. The chemotherapy regimens included etoposide/cisplatin, paclitaxel/cisplatin/bevacizumab, and paclitaxel/carboplatin. Among patients with confirmed chemotherapy regimens, 16 out of 19 received a combination of cisplatin with etoposide. Significant age differences were observed among the subtypes (*p* = 0.003). Patients with cervical NEC had a younger mean age (45.63 ± 10.58 year), whereas those with endometrial NEC were significantly older (59.86 ± 10.25 year). Patients with cervical NEC also had the highest proportion of early‐stage disease (65.9%, 27/41), whereas patients with endometrial NEC were more likely to present at advanced disease stages, with 57.1% (4/7) of patients diagnosed at stage III. Histologically, 53.8% (28/52) of the patients had MiNENs, and patients with ovarian NEC accounted for the highest proportion (75%, 3/4). Treatment modalities differed significantly (*p* < 0.001), with surgery combined with adjuvant therapy being the most common approach (48.1%, 25/52). Patients with cervical NEC mostly underwent surgery combined with radiotherapy and chemotherapy (53.7%, 22/41), whereas all patients with ovarian NEC underwent surgery combined with chemotherapy.

**TABLE 1:** Patient characteristics at the time of NEC diagnosis (*n* = 52).

Characteristics	Overall cohort (*n* = 52)	Patients with cervical NEC (*n* = 41)	Patients with endometrial NEC (*n* = 7)	Patients with ovarian NEC (*n* = 4)	*p* value
Age (years), mean (SD)	48.29 (11.48)	45.63 (10.58)	59.86 (10.25)	55.25 (8.10)	0.003
BMI (kg/m^2^), mean (SD)	23.35 (2.69)	23.21 (2.76)	24.15 (2.87)	23.39 (1.72)	0.702
FIGO stage					
I	31 (59.6)	27 (65.9)	2 (28.6)	2 (50.0)	0.258
II	5 (9.6)	4 (9.8)	1 (14.3)	0 (0.0)	
III	13 (25.0)	7 (17.1)	4 (57.12)	2 (50.0)	
IV	3 (5.8)	3 (7.3)	0 (0.0)	0 (0.0)	
Histology^a^					
MiNEN	28 (53.8)	23 (56.1)	2 (28.6)	3 (75.0)	0.550
Pure NEC	23 (44.2)	17 (41.5)	5 (71.4)	1 (25.0)	
Unknown	1 (1.9)	1 (2.4)	0 (0.0)	0 (0.0)	
Histology^b^					
HGNET	23 (44.2)	19 (46.3)	3 (42.9)	1 (25.0)	0.115
SCNEC	7 (13.5)	3 (7.3)	2 (28.6)	2 (50.0)	
LCNEC	22 (42.3)	19 (46.3)	2 (28.6)	1 (25.0)	
LNM					
Negative	36 (69.2)	31 (75.6)	3 (42.9)	2 (50.0)	0.109
Positive	13 (25.0)	8 (19.5)	4 (57.1)	1 (25.0)	
Unknown	3 (5.8)	2 (4.9)	0 (0.0)	1 (25.0)	
Treatment^c^					
CT	1 (1.9)	1 (2.4)	0 (0.0)	0 (0.0)	< 0.001
RT + CT	1 (1.9)	1 (2.4)	0 (0.0)	0 (0.0)	
Surgery	2 (3.8)	1 (2.4)	1 (14.3)	0 (0.0)	
Surgery + CT	6 (11.5)	2 (4.9)	0 (0.0)	4 (100.0)	
Surgery + CT + X	1 (1.9)	1 (2.4)	0 (0.0)	0 (0.0)	
Surgery + RT	1 (1.9)	0 (0.0)	1 (14.3)	0 (0.0)	
Surgery + RT + CT	25 (48.1)	22 (53.7)	3 (42.9)	0 (0.0)	
Surgery + X	12 (23.1)	10 (24.4)	2 (28.6)	0 (0.0)	
X	3 (5.8)	3 (7.3)	0 (0.0)	0 (0.0)	

*Note:* Values are presented as mean (SD) or number of patients (%).

Abbreviations: BMI, body mass index; CT, chemotherapy; FIGO, International Federation of Gynecology and Obstetrics; HGNET, high‐grade neuroendocrine tumor; LCNEC, large cell neuroendocrine carcinoma; LNM, lymph node metastasis; MiNEN, mixed neuroendocrine‐non‐neuroendocrine neoplasms; NEC, neuroendocrine carcinoma; RT, radiotherapy; SCNEC, small cell neuroendocrine carcinoma.

^a^ Histology is classified as MiNEN and pure NEC. MiNEN contains a neuroendocrine component alongside a distinct nonneuroendocrine carcinoma, while pure NEC consists entirely of neuroendocrine carcinoma.

^b^ Histology is classified by different morphological features of neoplastic cells.

^c^ Unknown adjuvant therapy methods or the entire treatment approach are represented by “X”.

Cervical NEC represented the predominant histological subtype (78.8%, 41/52; Table 1). Among these patients, 36.6% (15/41) presented with tumor diameters > 4 cm at diagnosis (Table 2). Adenocarcinoma was the most common type admixed with NEC (36.6%, 15/41). Among patients with cervical NEC, 67.6% (23/34) tested positive for HPV16 or HPV18, with HPV18 being the predominant type (17/24). Lymph node metastasis was the most common form of metastatic spread and was observed in 80% (8/10) of patients with metastasis, whereas visceral organ involvement was rare. As shown in Table 2, the cutoff value for NLR (range 1.03–13.88) is 2.32. The median values of PNI and LDH are 52.35 and 173 U/L, respectively.

**TABLE 2:** Patient characteristics at the time of diagnosis of cervical NEC (*n* = 41).

Characteristics	Values
BMI (kg/m^2^), mean (SD)	23.2 (2.8)
Age (year), mean (SD)	45.6 (10.6)
< 50	25 (61.0)
≥ 50	16 (39.0)
Histology^a^	
Pure NEC	17 (41.5)
MiNEN	23 (56.1)
Squamous cell carcinoma	6 (14.6)
Adenocarcinoma	15 (36.6)
Adenosquamous carcinoma	2 (4.9)
Unknown	1 (2.4)
Tumor size (cm)	
≤ 4	26 (63.4)
> 4	15 (36.6)
HPV	
HPV 18	16 (39.0)
HPV 16	6 (14.6)
HPV 16 + HPV 18	1 (2.4)
hrHPV Negative	2 (4.9)
hrHPV Positive	8 (19.5)
Untested	8 (19.5)
Surgery performed	
Yes	36 (87.8)
No	3 (7.3)
Unknown	2 (4.9)
Radiotherapy	
Yes	23 (56.1)
No	4 (9.8)
Unknown	14 (34.1)
Chemotherapy	
Yes	27 (65.9)
No	1 (2.4)
Unknown	13 (31.7)
NLR, mean (SD)	3.3 (2.3)
≤ 2.32	16 (39.0)
> 2.32	25 (61.0)
PNI, mean (SD)	51.9 (4.6)
≤ 52.35	20 (48.8)
> 52.35	21 (51.2)
LDH (U/L), mean (SD)	201.6 (212.6)
≤ 173	21 (51.2)
> 173	20 (48.8)

*Note:* Values are presented as mean (SD) or number of patients (%).

Abbreviations: BMI, body mass index; hrHPV, high‐risk human papilloma viruses; LDH, lactate dehydrogenase; MiNEN, mixed neuroendocrine‐non‐neuroendocrine neoplasms; NEC, neuroendocrine carcinoma; NLR, Neutrophil to Lymphocyte Ratio; PNI, Prognostic Nutritional Index.

^a^ Histology is classified as MiNEN and pure NEC. MiNEN is further categorized into MiNEN with squamous cell carcinoma, adenocarcinoma, and adenosquamous carcinoma.

### 3.2 IHC Features and the Ki‐67 Index

IHC evaluation revealed variable expression of neuroendocrine markers. Among the 46 cases with complete records, 15 (32.6%) were tested for two of the four key neuroendocrine markers (Syn, CgA, CD56, and NSE), 20 (43.5%) were tested for three markers, and 11 (23.9%) were tested for all four markers. Syn was positive in 95.3% (41/43) of patients, whereas CgA was positive in 72.7% (32/44) of patients (Table 3). Additional markers, including CD56 and NSE, further supported neuroendocrine differentiation, with positivity rates of 83.3% (25/30) and 70.6% (12/17), respectively. Figure S2 shows representative histologic and IHC images of cervical and ovarian NEC. Notably, these commonly used diagnostic markers for NEC were not uniformly positive across all patients, highlighting the role of these markers as adjuncts to the morphological diagnosis. The proliferative activity, assessed using the Ki‐67 index, was found to be high (> 50%) in 89.1% (41/46) of patients, although the Ki‐67 index exceeded 75% in only 23.9% (11/46) of patients. Incomplete testing for certain markers, such as CgA (15.4% untested), revealed variability in clinical diagnostic workflows (Table S1).

**TABLE 3:** Immunohistochemical characteristics in patients with NEC.

Characteristics	Overall (*n* = 52)		Characteristics	Overall (*n* = 52)	
	Tested (%)	Untested (%)		Tested (%)	Untested (%)
CgA			P16		
Negative	12 (27.3)	8 (15.4)	Negative	1 (2.4)	11 (21.2)
Positive	32 (72.7)		Positive	40 (97.6)	
Syn			P53		
Negative	2 (4.7)	9 (17.3)	Mutant	17 (39.5)	9 (17.3)
Positive	41 (95.3)		Wild type	26 (60.5)	
NSE			Ki‐67		
Negative	5 (29.4)	35 (67.3)	> 50%	41 (89.1)	6 (11.5)
Positive	12 (70.6)		≤ 50%	5 (10.9)	
CD56					
Negative	5 (16.7)	22 (42.3)			
Positive	25 (83.3)				

*Note:* All the cases are categorized by the expression of various markers and the percentage of cases either tested or untested for each marker.

Abbreviations: CgA, chromogranin A; NEC, neuroendocrine carcinoma; NSE, neuron‐specific enolase; Syn, synaptophysin.

### 3.3 Survival Outcomes and Prognostic Factors

**FIGURE 2:**
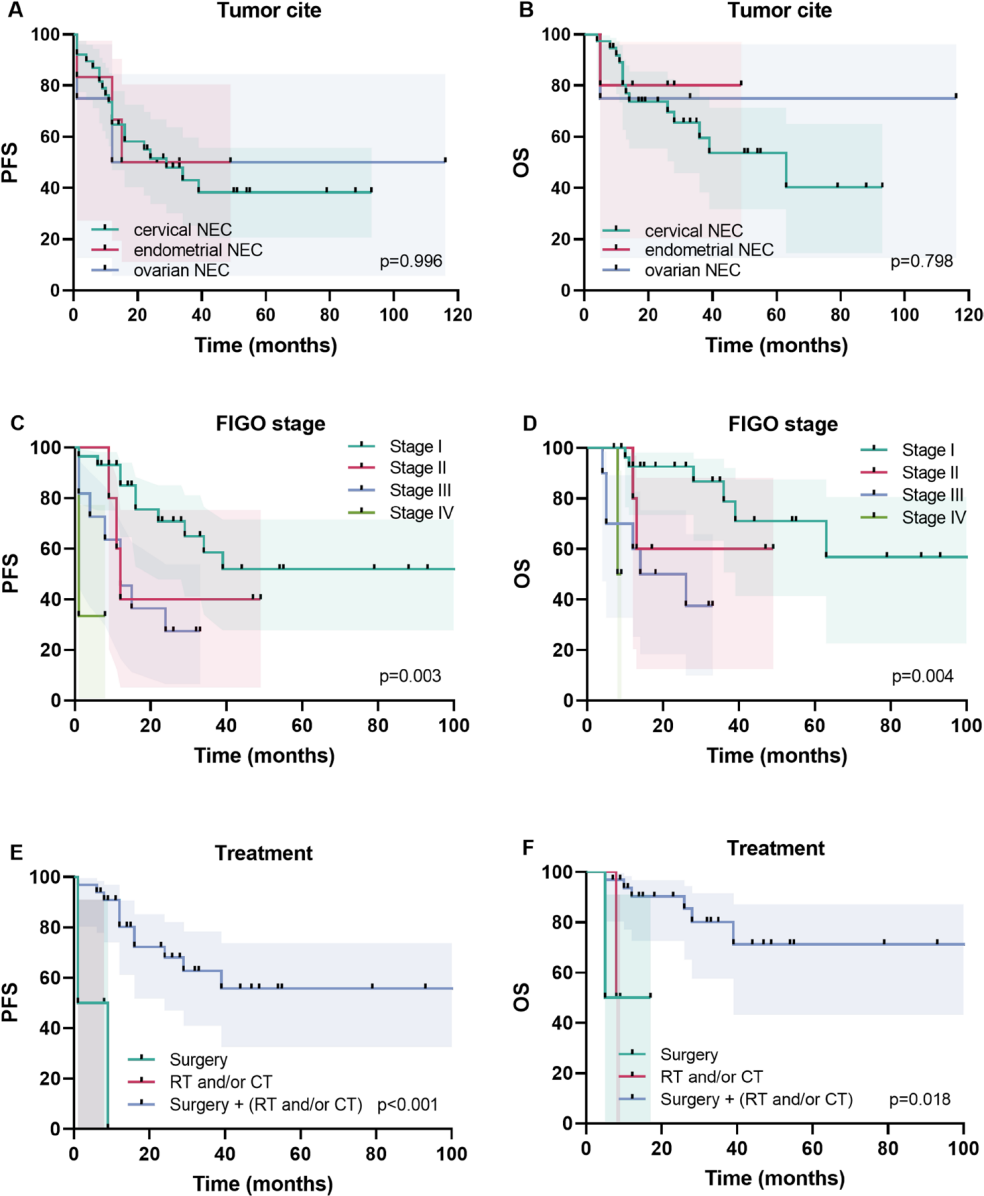
PFS (A, C, and E) and OS (B, D, and F) rates of patients with different tumor sites, FIGO stages, and treatment modalities. NEC, neuroendocrine carcinoma; OS, overall survival; PFS, progression‐free survival.

Survival analysis was conducted on 48 patients diagnosed with NEC of the gynecological tract. For patients with cervical NEC, the estimated PFS rates were 64.6% (95% CI: 46.9%–77.8%) at 1 year, 51.5% (95% CI: 33.7%–66.7%) at 2 year, and 38.3% (95% CI: 20.7%–55.6%) at 5 year. The OS rates were 80.1% (95% CI: 62.7%–90.0%) at 1 year, 73.7% (95% CI: 55.4%–85.4%) at 2 year, and 53.6% (95% CI: 31.7%–71.2%) at 5 year. The median PFS was 29 m for patients with cervical NEC, 32 m for patients with endometrial NEC, and 64 m for patients with ovarian NEC. The median OS for patients with cervical NEC was 63 m. The number of patients with endometrial NEC decreased from 5 at baseline to 4 at 5 m, with the last patient censored at 49 m. The number of patients with ovarian NEC decreased from 4 at baseline to 3 at 5 m, with the last patient censored at 116 m (Figure 2). No significant differences in PFS (*p* = 0.996) or OS (*p* = 0.798) were observed across the tumor sites. However, disease stage was significantly associated with prognosis. Patients with advanced‐stage disease exhibited markedly reduced PFS (*p* = 0.0008) and OS (*p* < 0.0004). In the overall cohort, surgery combined with adjuvant therapy (radiotherapy and/or chemotherapy) significantly improved PFS (*p* < 0.0001) and OS (*p* = 0.0107) (Figure 2). Among patients who underwent surgery alone, one patient with stage II cervical NEC experienced recurrence 9 m postoperatively without adjuvant therapy, whereas another patient with stage III endometrial NEC experienced rapid disease progression after surgery. Both patients who received primary radiochemotherapy had stage IV cervical NEC, which contributed to their poor outcomes.

**FIGURE 3:**
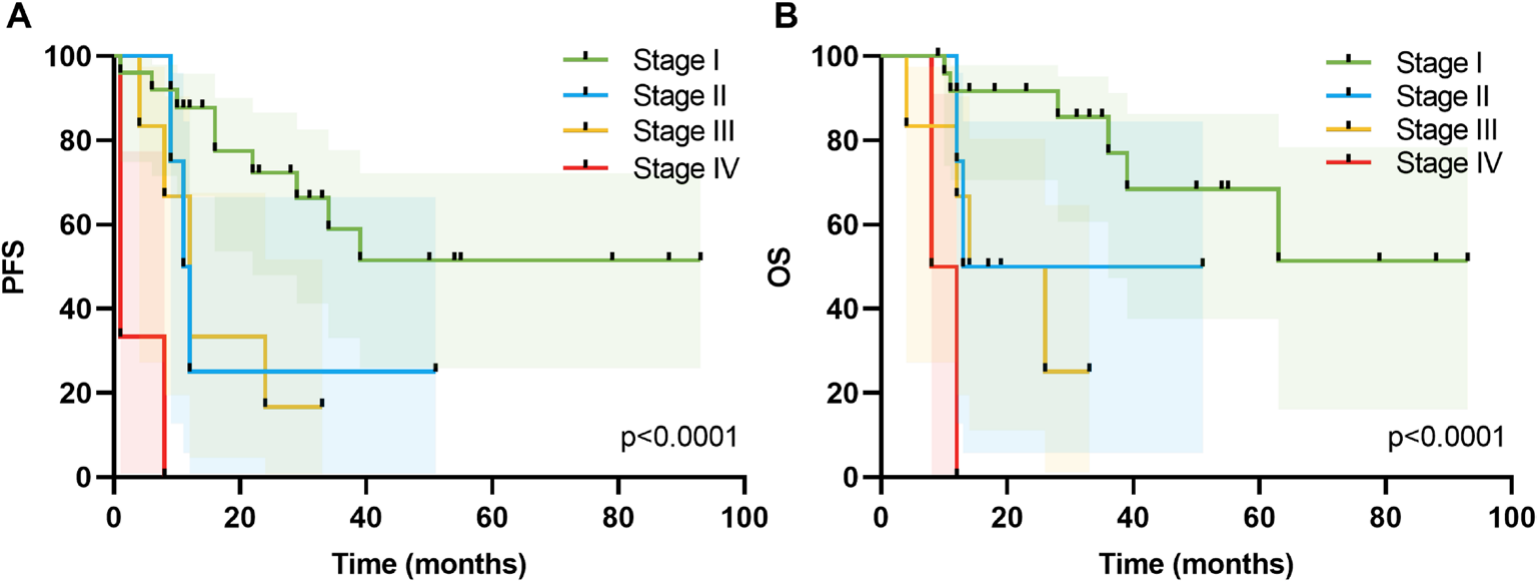
PFS (A) and OS (B) rates in patients with cervical NEC at different FIGO 2018 stages. FIGO, International Federation of Gynecology and Obstetrics; NEC, neuroendocrine carcinoma; OS, overall survival; PFS, progression‐free survival.

Figure 3 shows the PFS and OS curves for patients with cervical NEC, categorized by FIGO stages (I–IV), over a follow‐up period of up to 93 m. Stage I patients had a 5‐y PFS rate of 51.6% (95% CI: 26.0%–72.2%), whereas stage II patients had a significantly lower PFS rate of 25.0% (95% CI: 0.9%–66.5%) at 12 m. Stage III patients experienced a sharp decrease in PFS, with only 33.3% (95% CI: 4.6%–67.6%) remaining progression free at 12 m. Stage IV patients exhibited rapid disease progression, with a PFS rate of 33.3% (95% CI: 0.9%–77.4%) at 1 m and 0% at 8 m. The 2‐year OS rate of stage I patients was 91.7% (95% CI: 70.6%–97.8%), which decreased to 68.4% (95% CI: 37.6%–86.3%) at 5 year. In contrast, stage II patients had a 2‐year OS rate of 50.0% (95% CI: 5.8%–84.5%), with a median survival of 32 m. Stage III patients had a 2‐year OS rate of 50.0% (95% CI: 11.1%–80.4%), with a median survival of 20 m. Stage IV patients had a 1‐y OS rate of 50.0% (95% CI: 0.6%–91.0%), with no survival beyond 12 m. Stage‐stratified Kaplan–Meier curves for endometrial and ovarian NEC are provided in Figure S1 to illustrate site‐specific survival patterns based on limited sample sizes.

**FIGURE 4:**
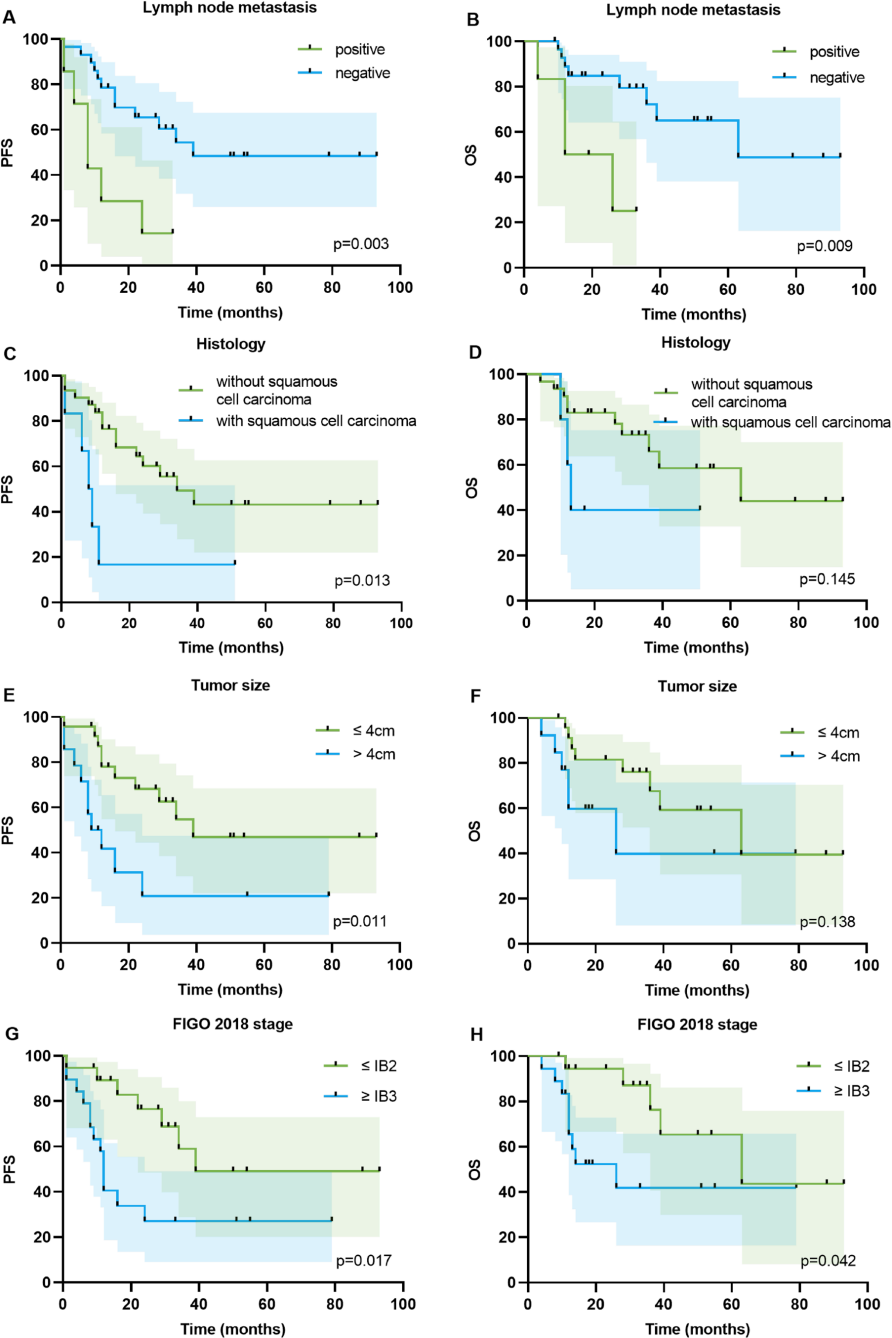
Comparison of PFS (A, C, E, and G) and OS (B, D, F, and H) rates with different factors. FIGO, International Federation of Gynecology and Obstetrics; OS, overall survival; PFS, progression‐free survival.

Univariate analysis revealed that factors such as FIGO stage ≥ IB3 (HR = 3.06, 95% CI: 1.22–7.68; *p* = 0.017), MiNEN with squamous cell carcinoma (HR = 7.03, 95% CI: 1.50–32.96; *p* = 0.013), tumor size > 4 cm (HR = 3.79, 95% CI: 1.36–10.61; *p* = 0.011), and lymph node metastasis (HR = 9.01, 95% CI: 2.07–39.26; *p* = 0.003) were significantly associated with worse PFS (Table 4). In the multivariate analysis, MiNEN with squamous cell carcinoma emerged as an independent prognostic factor for PFS (HR = 6.97, 95% CI: 1.60–30.31; *p* = 0.010). The survival curves are shown in Figure 4. In the univariate analysis for OS (Table 4), factors such as FIGO stage ≥ IB3 and lymph node metastasis were significant predictors of decreased OS. However, in the multivariate analysis, none of these factors remained independent risk factors for OS. The survival curves are presented in Figure 4.

**TABLE 4:** Prognostic factors for patients with cervical NEC.

Characteristics	No. of patients	PFS				No. of patients	OS			
		Univariate		Multivariate			Univariate		Multivariate	
		HR (95% CI)	*p* value	HR (95% CI)	*p* value		HR (95% CI)	*p* value	HR (95% CI)	*p* value
Age (years)										
< 50	24	1	—			25	1	—		
≥ 50	12	1.23 (0.47–3.24)	0.670			12	0.63 (0.20–1.97)	0.427		
LNM										
Negative	29	1	—	1	—	29	1	—	1	—
Positive	7	9.01 (2.07–39.26)	0.003	3.67 (1.00–13.46)	0.050	6	12.77 (1.91–85.43)	0.009	3.76 (0.77–18.47)	0.103
Histology										
MiNEN	21	1	—			20	1	—		
Pure NEC	15	0.50 (0.20–1.29)	0.150			15	0.68 (0.22–2.11)	0.495		
Histology^a^										
Without adenocarcinoma	23	1	—			22	1	—		
With adenocarcinoma	13	0.93 (0.35–2.46)	0.089			13	0.97 (0.29–3.24)	0.712		
Histology^b^										
Without squamous cell carcinoma	30	1	—	1	—	31	1	—		
With squamous cell carcinoma	6	7.03 (1.50–32.96)	0.013	6.97 (1.60–30.31)	0.010	5	3.78 (0.63–22.62)	0.145		
Tumor size										
≤ 4 cm	23	1	—	1	—	24	1	—		
> 4 cm	13	3.79 (1.36–10.61)	0.011	4.58 (0.76–27.49)	0.096	13	2.52 (0.74–8.55)	0.138		
HPV										
HPV 16	6	1	—			6	1	—		
HPV 18	16	0.40 (0.07–2.19)	0.244			16	0.29 (0.03–2.66)	0.235		
FIGO stage (2018)										
≤ IB2	18	1	—	1	—	19	1	—	1	—
≥ IB3	18	3.06 (1.22–7.68)	0.017	0.30 (0.04–2.11)	0.226	18	3.15 (1.04–9.52)	0.042	1.51 (0.36–6.39)	0.572
Ki‐67										
≤ 50%	4	1	—			4	1	—		
> 50%	28	1.39 (0.37–5.23)	0.623			28	0.92 (0.40–3.44)	0.915		
PNI										
≤ 52.35	19	1	—			19	1	—		
> 52.35	19	1.11 (0.43–2.84)	0.831			19	1.18 (0.08–1.53)	0.763		
NLR										
≤ 2.32	15	1	—			15	1	—		
> 2.32	23	0.74 (0.30–1.86)	0.525			23	0.60 (0.20–1.79)	0.360		
LDH (U/L)										
≤ 173	19	1	—			19	1	—		
> 173	19	0.89 (0.37–2.18)	0.804			19	0.50 (0.17–1.47)	0.209		

Abbreviations: CI, confidence interval; FIGO, International Federation of Gynecology and Obstetrics; HPV, human papillomavirus; HR, hazard ratio; LDH, lactate dehydrogenase; LNM, lymph node metastasis; MiNEN, mixed neuroendocrine‐non‐neuroendocrine neoplasms; NEC, neuroendocrine carcinoma; NLR, Neutrophil to Lymphocyte Ratio; OS, overall survival; PFS, progression‐free survival; PNI, Prognostic Nutritional Index.

^a^ Histology is classified as adenocarcinoma‐containing (MiNEN) or adenocarcinoma‐not‐containing (pure NEC or MiNEN).

^b^ Histology is classified as squamous cell carcinoma‐containing (MiNEN) or squamous‐not‐containing (pure NEC or MiNEN).

## 4 Discussion

The existing knowledge of gynecologic NECs stems largely from small case series and retrospective studies, emphasizing the need for more comprehensive data. This study contributes to the literature by examining the clinicopathological characteristics and survival outcomes of patients with gynecological NEC treated over a 10‐y period at a single institution. These findings reaffirm the aggressive nature and rarity of NEC, which is characterized by early metastasis and poor prognosis, particularly in cases of advanced‐stage disease [4]. Primary screening modalities for gynecological malignancies, such as ultrasound, CT, MRI, and tumor marker evaluation, have significantly improved the detection rates of various cancers [18]. However, NECs—particularly the endometrial subtype—often exhibit rapid progression, which may account for the challenges in diagnosing these tumors at early stages, leading to delayed treatment. Notably, HPV16/18 positivity was found in 69.7% of patients with cervical NEC, emphasizing the critical role of HPV testing in screening for cervical NEC. However, the absence of specific biomarkers or imaging features unique to NECs, particularly for ovarian and endometrial NECs, highlights an unmet clinical need for the development of targeted screening strategies to enhance early detection and improve outcomes for these aggressive tumors.

Cervical NEC was the predominant subtype in this study (78.8% of cases), and other reports have identified cervical NEC as the most common form of gynecologic NEC [3]. The stage‐specific outcomes in this study demonstrated that patients with stage I cervical NEC had a 5‐y OS rate of 68.4%, whereas no stage IV patients survived beyond 12 m. These findings are consistent with those of previous reports, which revealed rapidly decreasing survival rates with increasing disease stage. For example, a multicenter retrospective study from Japan reported 5‐y OS rates of 64.5%, 50.1%, 30.2%, and 3.4% for patients with stages I, II, III, and IV cervical NEC, respectively [19]. For patients with ovarian NEC, the SEER database indicates a 5‐y survival rate of 27.6% [20]. A retrospective study of 42 patients with endometrial NEC reported 5‐y OS rates of 88.9%, 100.0%, 46.7%, and 21.4% for patients with FIGO stage I, II, III, and IV disease, respectively [13]. The discrepancy in outcomes between this study and others may be partly due to evolving definitions and the diagnostic heterogeneity of NEC [3, 20]. Moreover, diagnostic challenges such as the misclassification of NECs, particularly ovarian NECs, where a misdiagnosis rate as high as 46% has been reported, could account for these variations [12].

A key challenge in analyzing survival outcomes is the inconsistent grading of tumors in clinical studies, where both low‐grade and high‐grade neuroendocrine tumors are often grouped together [20]. Low‐grade NETs generally exhibit less aggressive behavior, whereas NECs are highly aggressive, making it critical to distinguish between these subtypes in survival analyses. The inclusion of low‐grade tumors can result in an overestimation of survival rates for NEC patients. Our study excluded patients with well‐differentiated neuroendocrine tumors based on the diagnostic criteria of the 5th edition of the WHO Classification of Tumors of the Female Reproductive Organs, thereby enhancing the consistency of pathology [21]. Additionally, the survival rates for patients with MiNENs are often higher than those for patients with pure NECs, but this is not always clearly delineated in studies [12, 13].

The prognostic significance of mixed histology in gynecologic MiNENs remains a subject of debate [11, 12, 13, 22]. Our study identified MiNEN with squamous cell carcinoma as a potential high‐risk prognostic factor in patients with cervical NEC. However, this conclusion is limited by the retrospective design, small sample size, and single‐center nature of our cohort, all of which may introduce selection bias and overfitting. This finding contrasts with prior reports suggesting that mixed histology has no prognostic impact on patients with cervical NEC [11, 22] or that pure NEC histology is associated with poorer outcomes in patients with ovarian/endometrial NEC [12, 13]. Bellone et al. demonstrated significantly higher nonsilent mutation rates in NETs than in cervical adenocarcinoma based on tumor mutational burden analysis, with no significant difference observed between NETs and lesions with a squamous histology [16]. Taken together, these findings emphasize that the histologic composition—especially a squamous histology—influences both the therapeutic response and prognosis. This highlights the need for personalized management strategies and calls for further prospective studies to validate these results.

Many studies have investigated the prognostic value of inflammation‐based biomarkers, such as the NLR, Glasgow Prognostic Score (GPS), PNI, LDH, and Systemic Inflammation Response Index (SIRI), in predicting cancer outcomes [23, 24, 25, 26, 27]. However, our study did not identify a significant association between NLR, PNI, or LDH and the prognosis of cervical NEC. Since most of the patients were in early stage (75.7%), whether there will be different results in patients with advanced stages requires further investigation.

In terms of treatment, our study revealed that most patients, despite being diagnosed at an early stage, underwent surgery followed by adjuvant therapy, including chemotherapy and radiotherapy. Consistent with the literature, multimodal therapy, primarily surgery supplemented with chemotherapy or radiotherapy, serves as the mainstay of treatment for patients with early‐stage gynecologic NEC, regardless of the specific subtype of NEC [4, 5, 13, 28]. Cisplatin combined with etoposide is the most commonly used chemotherapy regimen [29]. However, treatment strategies for patients with gynecological NECs remain largely ineffective, with survival outcomes still poor across all stages of the disease [13, 19, 20]. As demonstrated by our study, the prognosis remains poor even in patients diagnosed with early‐stage disease receiving multimodal therapy. The development of more effective and targeted therapeutic strategies is urgently needed.

We included a diverse cohort of patients with ovarian, endometrial, and cervical carcinomas, which allowed for a more thorough understanding of the clinical features of NECs. Adherence to the 5th WHO criteria (high‐grade neuroendocrine tumors only) and the reclassification of cervical NECs using the 2018 FIGO staging system enhanced diagnostic homogeneity, revealing significant survival disparities. Notably, we identified MiNEN with squamous cell carcinoma as an independent prognostic factor for PFS in patients with cervical NEC (HR = 6.97, *p* = 0.010), a novel finding that warrants further validation. Our multisite design also highlighted distinct clinical profiles: endometrial NECs primarily presented at advanced stages, whereas cervical NECs often presented at earlier stages with symptoms. Furthermore, single‐institution standardization minimized treatment heterogeneity, thus strengthening internal validity.

However, this study has several limitations. First, the rarity of gynecologic NECs resulted in a small cohort, particularly for the ovarian and endometrial subtypes, which limits statistical power, produces wide confidence intervals, and precludes robust subgroup analyses. Second, the retrospective, single‐center design may introduce selection bias and treatment variability, reducing the generalizability of the findings. These factors limit the robustness of prognostic analyses, and our results should be interpreted as descriptive and hypothesis generating rather than confirmatory. Additionally, some cases lacked a complete IHC panel, reflecting real‐world diagnostic variability. Future multicenter prospective studies incorporating standardized pathology reviews, molecular profiling, and uniform treatment protocols are needed to validate these observations and guide evidence‐based management of gynecologic NECs.

In conclusion, this study highlights the aggressive clinical course of gynecological NECs, even at early stages. Our single‐institution, multisite analysis suggests that multimodal therapy remains the predominant approach but yields limited survival benefit. These findings, together with the identification of MiNEN with squamous cell carcinoma as a potential adverse prognostic factor, provide real‐world evidence to inform future clinical management. Future multicenter prospective studies incorporating molecular profiling and standardized treatment protocols are needed to validate these observations and explore the best therapeutic strategies.

## Author Contributions

Yedan Ren: Data curation; Visualization; Formal analysis; Writing – original draft. Sen Li: Data curation; Visualization; Formal analysis; Funding acquisition. Simin Xiang: Data curation; Formal analysis. Junfen Xu: Writing – review and editing; Supervision; Funding acquisition; Project administration.

## Funding

This work was supported by the National Natural Science Foundation of China (general program, nos.: 82072855 and 82472891); the Fund for Young Scientists (Program C, grant no.: 82203621) and the Zhejiang Provincial Natural Science Foundation of China (key program, grant no.: LZ24H160001).

## Ethics Statement

The study was approved by the Ethics Committee of Women's Hospital, Zhejiang University School of Medicine (IRB‐20230384‐R).

## Consent

Due to the retrospective design of this study, the Ethics Committee granted a waiver of informed consent.

## Conflicts of Interest

The authors declare no conflicts of interest.

## Supporting information


**Figure S1:** Kaplan–Meier curves for PFS (A, C) and OS (B, D) stratified by FIGO stage in endometrial NEC (*n* = 6) and ovarian NEC (*n* = 4). FIGO, international federation of gynecology and obstetrics; PFS, progression‐free survival; OS, overall survival.

**Figure S2:** Representative histological and IHC features of gynecologic NECs. Panels (A), (D), (G), and (J) are from a patient with cervical SCNEC. Panels (B), (E), (H), and (K) are from a patient with ovarian SCNEC admixed with high‐grade serous carcinoma. The images specifically show the SCNEC component. Panels (C), (F), and (I) are from a patient with endometrial LCNEC. H&E staining of cervical SCNEC (A) and ovarian SCNEC (B) shows cells with hyperchromatic nuclei, scant cytoplasm, and abundant mitotic activity. H&E staining of endometrial LCNEC (C) shows cells with moderate amounts of cytoplasm and large nuclei with coarse chromatin and prominent nucleoli. Cervical SCNEC shows diffuse positivity for Syn (D) and CD56 (J), with focal positivity for CgA (G). Ovarian SCNEC shows diffuse positivity for Syn (E), CgA (H), and NSE (K). Endometrial LCNEC shows positivity for Syn (F) but negativity for CgA (I). Scale bars: 100 μm. CgA, chromogranin A; H&E, hematoxylin and eosin; IHC, immunohistochemistry; LCNEC, large‐cell neuroendocrine carcinoma; NSE, neuron‐specific enolase; SCNEC, small‐cell neuroendocrine carcinoma; Syn, synaptophysin.

**Table S1:** Summary of IHC markers used in representative NEC cases.

## Data Availability

The data that support the findings of this study are available on request from the corresponding author. The data are not publicly available due to privacy or ethical restrictions.
