# Supplementary material for: A Retrospective Study on the Clinicopathological Characteristics and Prognostic Analysis of Gynecologic Neuroendocrine Carcinoma
Source: Cancer Med. 2025 Dec 31;15(1):e71488. doi: 10.1002/cam4.71488 (PMC12755394; doi:10.1002/cam4.71488)
Supplement: Supplementary file 1 — Figure S1: Kaplan–Meier curves for PFS (A, C) and OS (B, D) stratified by FIGO stage in endometrial NEC (n = 6) and ovarian NEC (n = 4). FIGO, international federation of gynecology and obstetrics; PFS, progression‐free survival; OS, overall survival. [file CAM4-15-e71488-s002.pdf]

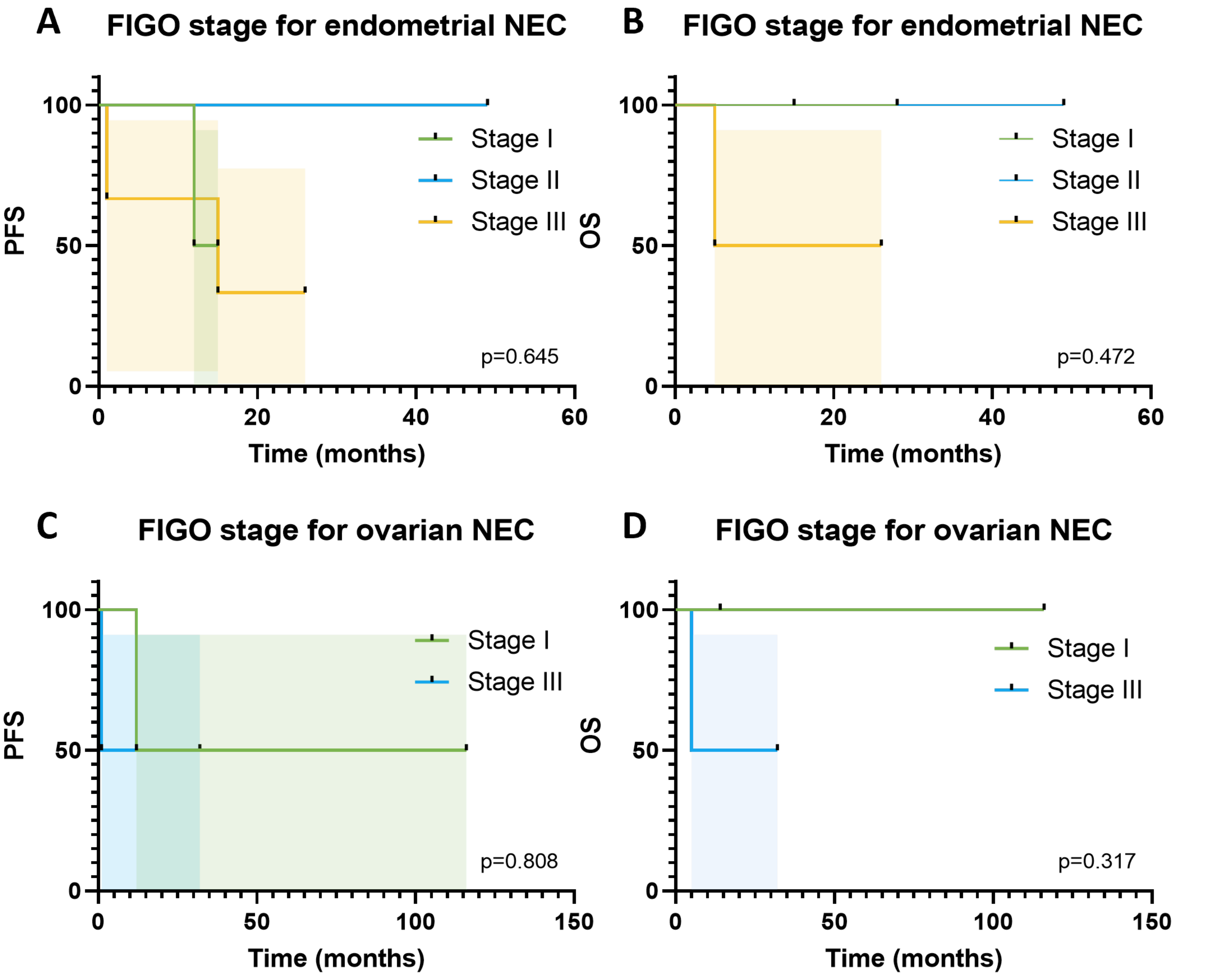

A
FIGO stage for endometrial NEC
PFS
100
50
0
0
20
40
60
Time (months)
Stage I
Stage II
Stage III
p=0.645
B
FIGO stage for endometrial NEC
OS
100
50
0
0
20
40
60
Time (months)
Stage I
Stage II
Stage III
p=0.472
C
FIGO stage for ovarian NEC
PFS
100
50
0
0
50
100
150
Time (months)
Stage I
Stage III
p=0.808
D
FIGO stage for ovarian NEC
OS
100
50
0
0
50
100
150
Time (months)
Stage I
Stage III
p=0.317
