# Supplementary figures and images for: A Retrospective Study on the Clinicopathological Characteristics and Prognostic Analysis of Gynecologic Neuroendocrine Carcinoma
Source: Cancer Med. 2025 Dec 31;15(1):e71488. doi: 10.1002/cam4.71488 (PMC12755394; doi:10.1002/cam4.71488)

## Cervical NEC

## Ovarian NEC

## Endometrial NEC

H&E

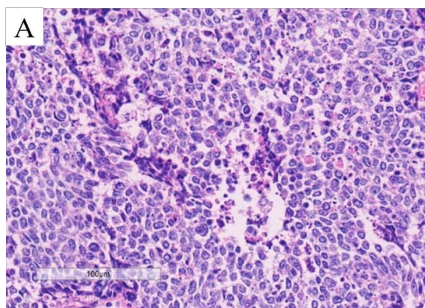

H&E

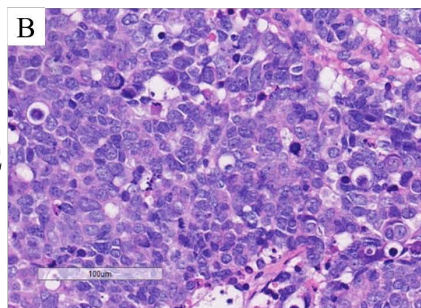

H&E

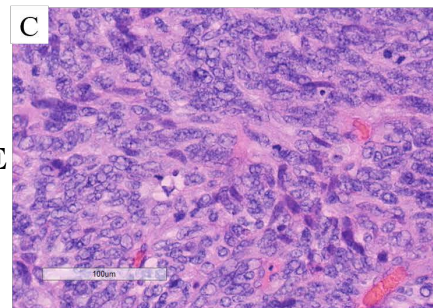

Syn

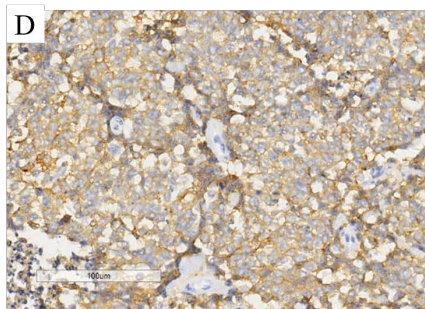

Syn

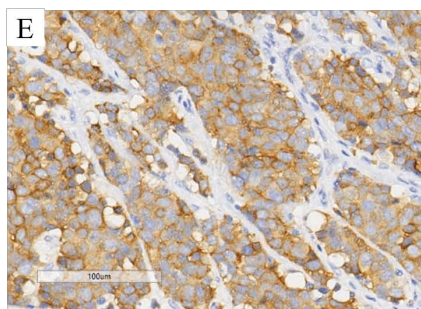

Syn

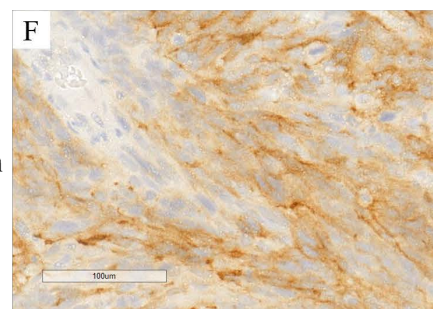

CgA

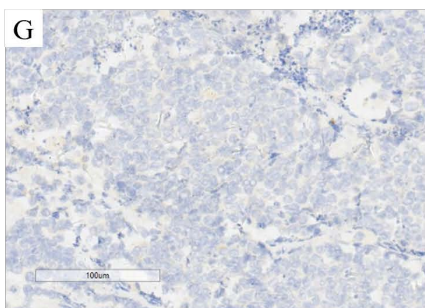

CgA

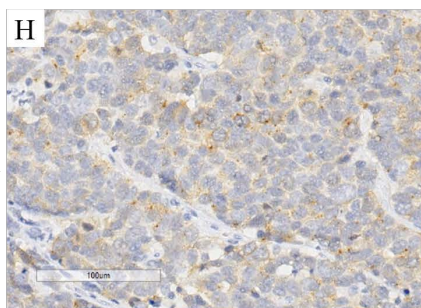

CgA

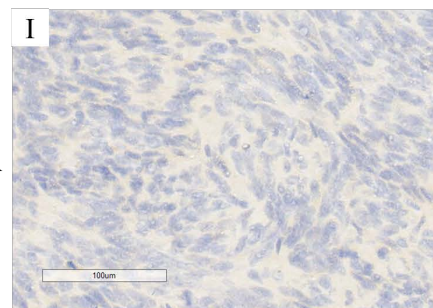

CD56

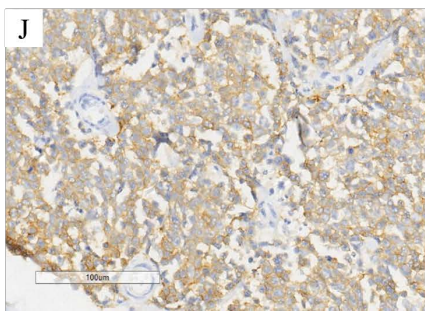

NSE

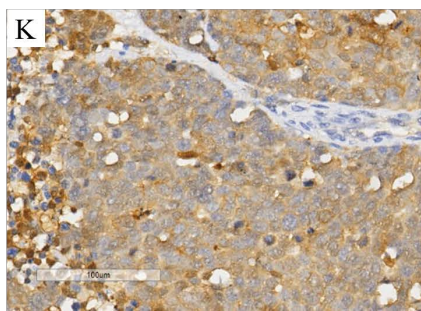

Supplement: Supplementary file 2 — Figure S2: Representative histological and IHC features of gynecologic NECs. Panels (A), (D), (G), and (J) are from a patient with cervical SCNEC. Panels (B), (E), (H), and (K) are from a patient with ovarian SCNEC admixed with high‐grade serous carcinoma. The images specifically show the SCNEC component. Panels (C), (F), and (I) are from a patient with endometrial LCNEC. H&E staining of cervical SCNEC (A) and ovarian SCNEC (B) shows cells with hyperchromatic nuclei, scant cytoplasm, and abundant mitotic activity. H&E staining of endometrial LCNEC (C) shows cells with moderate amounts of cytoplasm and large nuclei with coarse chromatin and prominent nucleoli. Cervical SCNEC shows diffuse positivity for Syn (D) and CD56 (J), with focal positivity for CgA (G). Ovarian SCNEC shows diffuse positivity for Syn (E), CgA (H), and NSE (K). Endometrial LCNEC shows positivity for Syn (F) but negativity for CgA (I). Scale bars: 100 μm. CgA, chromogranin A; H&E, hematoxylin and eosin; IHC, immunohistochemistry; LCNEC, large‐cell neuroendocrine carcinoma; NSE, neuron‐specific enolase; SCNEC, small‐cell neuroendocrine carcinoma; Syn, synaptophysin. [file CAM4-15-e71488-s003.pdf]
