# Supplementary material for: A Retrospective Study on the Clinicopathological Characteristics and Prognostic Analysis of Gynecologic Neuroendocrine Carcinoma
Source: Cancer Med. 2025 Dec 31;15(1):e71488. doi: 10.1002/cam4.71488 (PMC12755394; doi:10.1002/cam4.71488)
Supplement: Supplementary file 3 — Table S1: Summary of IHC markers used in representative NEC cases. [file CAM4-15-e71488-s001.docx]

| **Supplementary Table S1. Summary of IHC markers used in representative NEC cases** | | | |
| --- | --- | --- | --- |
| **Case ID** | **Primary Site** | **Histological Type** | **Markers Used** |
| 1 | Cervix | MiNEN | Ki67, P16, CEA, CK-H, CK-L, P63, Syn, CgA, NSE, TTF1 |
| 2 | Cervix | MiNEN | D2-40, SOX2, EGFR, Cam5.2, P40, P63, P16, P53, Ki-67, CgA, SYN, CD56, NSE |
| 3 | Cervix | MiNEN | P16, ER, PAX8, WT-1, CA125, CEA, CD56, Syn, CgA, Ki-67, TTF-1, S-100, IMP-3, D2-40, PR, P53, CK-LMW |
| 4 | Cervix | Pure NEC | CK(-), CK8/18, EMA, HMB45, SOX-10, SYN, CgA, Vimentin, LCA, Ki-67, CD10, CK7, Desmin, S-100, BRG1, CD34, CD56, CD99, P16, TTF-1, P63, INI-1, CK-LMW, PAX8, Cam5.2 |
| 5 | Cervix | MiNEN | IMP-3, ER, PR, P53, CK-LMW, P16, Ki-67, LCA, SYN, CgA, CK, PAX8, E-cadherin |
| 6 | Cervix | Pure NEC | D2-40, SOX2, Cam5.2, P40, P63, P16, P53, Ki-67, SYN, CD56, CgA, GATA-3, CK |
| 7 | Cervix | Pure NEC | D2-40, SOX2, EGFR, P40, P63, P16, P53, Ki-67, CgA, SYN, CK, CK5/6, Cam5.2, CD56, NSE, PAX8, CK8/18 |
| 8 | Ovary | MiNEN | PAX8, MUC-6, SATB2, ER, PR, Mucin5AC, CK7, CK20, Ki-67, CEA, CDX2, P53, SYN, CgA |
| 9 | Ovary | MiNEN | P16, Ber-EP4, ER, PR, CA125, PAX8, WT1, Ki-67, P53, SYN, CgA, TTF-1, NSE, CD56, BRG1 |
| 10 | Endometrium | MiNEN | PMS2, MSH6, MLH1, MSH2, PAX2, PTEN, IMP-3, P16, ER, PR, Ki-67, P53, WT1, Vimentin, SYN, CgA, CD56 |
| 11 | Endometrium | Pure NEC | PMS2, MSH6, MLH1, MSH2, PAX2, PTEN, IMP-3, P16, ER, PR, Ki-67, P53, CK, SYN, CgA, Vimentin, CD10, CyclinD1 |
| This table lists the IHC markers applied in each case as part of routine diagnostic workup. The cases represent cervical NEC (cases 1-7), ovarian NEC (cases 8-9) and endometrial NEC (cases 10-11). | | | |
